# High prevalence of *Toxoplasma gondii* antibodies in dogs in Veracruz, Mexico

**DOI:** 10.1186/s12917-014-0191-x

**Published:** 2014-08-20

**Authors:** Cosme Alvarado-Esquivel, Dora Romero-Salas, Anabel Cruz-Romero, Zeferino García-Vázquez, Álvaro Peniche-Cardeña, Nelly Ibarra-Priego, Concepción Ahuja-Aguirre, Adalberto A Pérez-de-León, Jitender P Dubey

**Affiliations:** 1 Biomedical Research Laboratory, Faculty of Medicine and Nutrition, Juárez University of Durango State, Avenida Universidad S/N, Durango, 34000, Mexico; 2 Laboratorio de Parasitología, Facultad de Medicina Veterinaria y Zootecnia, Universidad Veracruzana, Circunvalación y Yáñez S/N, Xapala 91710, Veracruz, Mexico; 3 Centro Nacional de Investigación Disciplinaria en Parasitología Veterinaria, INIFAP. Carretera Federal Cuernavaca-Cuautla No. 8534, Jiutepec, C.P. 6225, Morelos, México; 4 United States Department of Agriculture, Agricultural Research Service, Knipling-Bushland U.S. Livestock Insects Research Laboratory, 2700 Fredericksburg Road, Kerrville 78028, Texas, USA; 5 United States Department of Agriculture, Agricultural Research Service, Beltsville Agricultural Research Center. Animal Parasitic Diseases Laboratory, Building 1001, Beltsville 20705-2350, Maryland, USA; 6 Laboratorio de Investigación Biomédica, Facultad de Medicina y Nutrición, Avenida Universidad S/N, Durango, 34000, Dgo, Mexico

**Keywords:** Toxoplasma gondii, Dogs, Modified agglutination test, Antibodies, Prevalence, Mexico

## Abstract

**Background:**

Little is known concerning the prevalence of *Toxoplasma gondii* infection in dogs in Mexico. Here, we investigated antibodies to *T. gondii* and associated risk factors in 101 dogs from an animal shelter in Veracruz State, Mexico. Canine sera were assayed for *T. gondii* IgG antibodies by using the modified agglutination test (MAT, cut off 1:25).

**Results:**

Sixty eight (67.3%) of 101 dogs were seropositive with titers of 1:25 in 16, 1:50 in 8, 1:100 in 9, 1:200 in 10, 1:400 in 10, 1:800 in 10, 1:1600 in 3, and 1:3200 or higher in 2. None of the dogs’ characteristics studied including age, sex, breed, and history of deworming, rabies vaccination and contact with cats was associated with seroprevalence of *T. gondii* infection.

**Conclusion:**

Using the dogs as sentinel animals, the results indicate high contamination with *T. gondii* of the environment in Veracruz, Mexico. Results have public health implications, and further studies in Veracruz should be conducted to establish the sources of environmental contamination with *T. gondii* and to determine optimal preventive measures against *T. gondii* infection in humans.

## Background

*Toxoplasma gondii* causes infections in most animals worldwide, including dogs [1]. Infection with *T. gondii* in dogs is usually asymptomatic but cases of severe clinical toxoplasmosis in dogs have been reported worldwide, including 1 report from Mexico [1]–[3]. Infection with *T. gondii* in dogs is also of epidemiological importance. The prevalence of *T. gondii* infection in dogs may reflect the magnitude of parasite contamination in their environment. For this reason dogs have been used as sentinel animal for *T. gondii* infection because of their close contact with people [4]. Dogs can also act as mechanical vectors for *T. gondii* oocysts because of their habit of eating cat feces and also rolling over in cat excreta [5]. If they ingest feces of infected cats, some of the oocysts can pass unchanged through the dog intestine and appear in feces; viable *T. gondii* oocysts have been detected in feces of naturally infected dogs in Europe [6] when they do so. In addition, dog meat is used for human consumption in some countries and viable *T. gondii* has been isolated from tissues of asymptomatic dogs [7].

Worldwide reports of *T. gondii* prevalence in dogs have been summarized [1],[3]. The objective of the present investigation was to determine the seroprevalence of *T. gondii* infection in dogs from an animal shelter in Veracruz, an eastern state of Mexico. In addition, the association of *T. gondii* seropositivity with general characteristics of the dogs was also investigated. There are only 3 previous reports of *T. gondii* prevalence in dogs from Mexico, and they are from different region than studied here [8]–[10].

## Methods

### Dogs surveyed

For the present study 101 dogs (*Canis familiaris*) that were in the animal shelter of the Medellín municipality in Veracruz State, Mexico from February to May 2013 were studied. The animal shelter holds stray dogs and unwanted pet dogs abandoned by the owners for adoption. The Medellín municipality is located in the Sotavento region (19°03’N 96°09 W) which has an altitude of 52 meters above sea level, a warm-humid climate, a mean annual rainfall of 1,417.8 mm, and a mean annual temperature of 25.3°C. Dogs are kept outdoors during the day in a yard with soil flooring, and indoors at nights. Most (88.1%) dogs had received deworming medication and some (32.7%) dogs had been vaccinated against rabies. A minority of dogs had had contact with cats (11.9%). Age in dogs ranged from 0.5 to > 4 years. Thirty seven (36.6%) dogs were males and 64 (63.4%) were females. A questionnaire was used to record the general characteristics of the dogs including age, sex, and breed (pure or mixed).

### Ethical aspects

This study was approved by the Bioethics and Animal Welfare Commission of the Facultad de Medicina Veterinaria y Zootecnia of Universidad Veracruzana, and consent was obtained from the owner of the animal shelter.

### Laboratory tests

Sera of dogs were obtained and stored at −20°C until assayed. Dog sera were tested for *T. gondii* IgG antibodies using 2-fold serial dilutions from 1:25 to 1:3,200 with the modified agglutination test (MAT) as described by Dubey and Desmonts [11]. A titer of 1:25 was used as cut off for seropositivity in MAT.

### Statistical analysis

Results were analyzed with the aid of the software Epi Info version 7 (Centers for Disease Control and Prevention: http://wwwn.cdc.gov/epiinfo/ and SPSS version 15.0 (SPSS Inc. Chicago, Illinois). For comparison of the frequencies among the groups, the Pearson’s chi-squared test, and when indicated the Fisher exact test, were used. Multivariable analysis (logistic regression analysis with the Enter method) was used to assess the association between the dogs’ characteristics and *T. gondii* seropositivity. The dependent variable was seropositivity by MAT for an individual animal. Independent variables included in the multivariable analysis were age, sex, breed, and history of deworming, rabies vaccination, and contact with cats. The Hosmer-Lemeshow goodness of fit test was used to assess the fitness of the regression model. Statistical significance was set at a *P* value of < 0.05.

## Results

IgG antibodies to *T. gondii* were detected in 68 (67.3%) of 101 dogs with titers of 1:25 in 16, 1:50 in 8, 1:100 in 9, 1:200 in 10, 1:400 in 10, 1:800 in 10, 1:1600 in 3, and 1:3200 or higher in 2.

General characteristics of the dogs and their relation with *T. gondii* seropositivity are shown in Table 1. Seroprevalence of *T. gondii* infection in dogs did not vary with age, sex, breed, and history of deworming, vaccination against rabies, and contact with cats. Further analysis by logistic regression did not show an association of the dogs’ characteristics and *T. gondii* seropositivity. The result of the Hosmer-Lemeshow test was 8 (*P* = 0.84), indicating an acceptable fit of our regression model.

**Table 1:** **General characteristics of the 101 dogs studied and seroprevalence of** ***T. gondii*** **infection**

		**Seroprevalence of** ***T. gondii*** **infection**		***P*** **value**
**Characteristics**		**No.**	**%**	
Age (years)				
0.5-1	47	35	74.5	0.31
1.1-2	26	15	57.7	
>2	28	18	64.3	
Sex				
Male	37	25	67.6	0.96
Female	64	43	67.2	
Breed				
Pure	37	28	75.7	0.17
Mixed	64	40	62.5	
Deworming				
Yes	89	59	66.3	0.74
No	12	9	75	
Vaccinated against rabies				
Yes	33	25	75.8	0.2
No	68	43	63.2	
Contact with cats				
Yes	12	7	58.3	0.52
No	89	61	68.5	

Further comparison between dogs with high (≥1:800) MAT titers and dogs with low (1:25–1:400) MAT titers did not show differences in their characteristics including age, sex, breed, and history of deworming, vaccination against rabies, and contact with cats (Table 2).

**Table 2:** **Correlation of high (≥1:800) MAT titers and general characteristics of the 68** ***T. gondii*-positive dogs studied**

		**Frequency of high MAT titers**		***P*** **value**
**Characteristics**	**No. of dogs**	**No.**	**%**	
Age (years)				
0.5-1	35	6	17.1	0.47
1.1-2	15	5	33.3	
>2	18	4	22.2	
Sex				
Male	25	3	12	0.22
Female	43	12	27.9	
Breed				
Pure	40	10	25	0.56
Mixed	28	5	17.9	
Deworming				
Yes	59	13	22	1
No	9	2	22.2	
Vaccinated against rabies				
Yes	25	7	28	0.38
No	43	8	18.6	
Contact with cats				
Yes	7	1	14.3	1
No	61	14	23	

## Discussion

In the present study, a high (67.3%) seroprevalence of *T. gondii* infection was found in dogs from an animal shelter in Veracruz, Mexico. As mentioned in introduction, there are 3 previous reports on the seroprevalence of infection with *T. gondii* in dogs in Mexico. Two studies performed in dogs in the northern Mexican city of Durango with the same MAT as we used in the present study reported seroprevalences of 45.3% [9] and 51.5% [8] of *T. gondii* infection. The third study performed in dogs in the southern Mexican state of Oaxaca reported a 61.7% seroprevalence of *T. gondii* by using an indirect ELISA [10]. Differences in the seroprevalences among the studies might be explained by environmental differences between the regions studied. Dogs in Veracruz State were raised in a warm humid climate while dogs in Durango were raised in a dry climate. The seroprevalence reported in dogs in Oaxaca (61.7%) is nearly as high as the seroprevalence found in dogs in Veracruz. However, comparison of the seroprevalence in dogs in Oaxaca and Veracruz should be interpreted with care since different serological tests (ELISA versus MAT) were used. The MAT detects antibodies against surface antigens because whole tachyzoites are used whereas soluble antigens are used in the ELISA, which detects antibodies directed against those fractions.

None of the general characteristics of dogs including age, sex, breed, history of deworming, vaccination against rabies and contact with cats were associated with *T. gondii* seropositivity in the present work. In a previous study in dogs in Durango City, Mexico the seroprevalence of *T. gondii* infection did not increase with age [8]. In contrast, an increase in seroprevalence of *T. gondii* infection with age was reported in dogs in China [12], and Brazil [13]. It is possible that differences in environmental characteristics among the countries might explain the differences in prevalence of *T. gondii* infection in dogs. Clinical congenital toxoplasmosis is considered rare in dogs [1]. However, there are no estimates of rates of asymptomatic transplacental or lactogenic transmission of *T. gondii* in dogs. Transcolostral antibodies in animals generally disappear by 3 months of post natal life [1]. The high seroprevalence (74.5%) in the youngest (0.5-1 year old) dogs indicates postnatal early exposure to *T. gondii* in dogs in Veracruz. The lack of association of *T. gondii* seropositivity with breed and sex is probably overshadowed by high exposure to *T. gondii* in dogs at very young age.

The high prevalence of *T. gondii* infection found in dogs in the shelter might be explained by the presence of several contributing factors for infection including: 1) predation of rodents; rats existed in the area and dogs probably hunted them; 2) eating garbage; many dogs in the shelter were rescued from streets and roads where they ate from garbage, and might have hunted birds and small mammals; 3) consumption of untreated water and leftovers that might have been contaminated/infected with *T. gondii*.

## Conclusions

Dogs in the Mexican state of Veracruz had very high *T. gondii* seroprevalence. This is indicative of high environmental contamination with *T. gondii* in Veracruz, Mexico. Results have public health implications including an important risk for *T. gondii* infection in humans in the region, and the risk for morbidity and mortality due to toxoplasmosis. Further studies in Veracruz should be conducted to establish the sources of environmental contamination with *T. gondii* and to determine optimal preventive measures against *T. gondii* infection in humans.

## Competing interests

The authors declare that they have no competing interests.

## Authors’ contributions

CAE performed the laboratory tests, data analysis, and wrote the manuscript. DRS designed the study protocol, obtained the blood samples and general data of the dogs, and analyzed the results. ZGV analyzed the results. ACR, NIP, CAA and APC obtained the blood samples and general data of the dogs, and analyzed the results. DRS and AAPL performed the data analysis and helped in the writing of the manuscript. JPD analyzed the results and wrote the manuscript. All authors read and approved the final version of the manuscript.

## References

[B1] Dubey JP Toxoplasmosis of animals and humans 2010 CRC Press, Boca Raton, Florida

[B2] de Aluja AS Toxoplasmosis: estudio anatomo-patológico de un caso en perro Veterinaria 1971 1 9 12

[B3] Dubey JP Beattie CP Toxoplasmosis of animals and man 1988 CRC Press, Boca Raton, Florida

[B4] Salb AL Barkema HW Elkin BT Thompson RC Whiteside DP Black SR Dubey JP Kutz SJ Dogs as sources and sentinels of parasites in humans and wildlife, northern Canada Emerg Infect Dis 2008 14 60 63 doi:10.3201/eid1401.071113 10.3201/eid1401.071113 18258078 PMC2600154

[B5] Frenkel JK, Lindsay DS, Parker BB: **Dogs as potential vectors of*****Toxoplasma gondii*****.** *Am J Trop Med Hyg* 1995, **53:**226.

[B6] Schares G, Pantchev N, Barutzki D, Heydorn AO, Bauer C, Conraths FJ: **Oocysts of*****Neospora caninum*****,*****Hammondia heydorni*****,*****Toxoplasma gondii*****and*****Hammondia hammondi*****in faeces collected from dogs in Germany.** *Int J Parasitol* 2005, **35:**1525–1537. 10.1016/j.ijpara.2005.08.008 16197949

[B7] El Behairy AM, Choudhary S, Ferreira LR, Kwok OC, Hilali M, Su C, Dubey JP: **Genetic characterization of viable*****Toxoplasma gondii*****isolates from stray dogs from Giza, Egypt.** *Vet Parasitol* 2013, **193:**25–29. doi:10.1016/j.vetpar.2012.12.007. 10.1016/j.vetpar.2012.12.007 23333072

[B8] Dubey JP, Alvarado-Esquivel C, Liesenfeld O, Herrera-Flores RG, Ramírez-Sánchez BE, González-Herrera A, Martínez-García SA, Bandini LA, Kwok OC: ***Neospora caninum*****and*****Toxoplasma gondii*****antibodies in dogs from Durango City, Mexico.** *J Parasitol* 2007, **93:**1033–1035. doi:10.1645/GE-1281R.1. 10.1645/GE-1281R.1 18163336

[B9] Dubey JP, Velmurugan GV, Alvarado-Esquivel C, Alvarado-Esquivel D, Rodríguez-Peña S, Martínez-García S, González-Herrera A, Ferreira LR, Kwok OC, Su C: **Isolation of*****Toxoplasma gondii*****from animals in Durango, Mexico.** *J Parasitol* 2009, **95:**319–322. doi:10.1645/GE-1874.1. 10.1645/GE-1874.1 18925790

[B10] Cedillo-Peláez C, Díaz-Figueroa ID, Jiménez-Seres MI, Sánchez-Hernández G, Correa D: **Frequency of antibodies to*****Toxoplasma gondii*****in stray dogs of Oaxaca, México.** *J Parasitol* 2012, **98:**871–872. 10.1645/GE-3095.1. 10.1645/GE-3095.1 22360636

[B11] Dubey JP, Desmonts G: **Serological responses of equids fed*****Toxoplasma gondii*****oocysts.** *Equine Vet J* 1987, **19:**337–339. 10.1111/j.2042-3306.1987.tb01426.x 3622463

[B12] Li Y, Liu Q, Li S, Wei F, Jin H, Yang M: **Seroprevalence of*****Toxoplasma gondii*****infection in dogs in Jiangsu Province, eastern China.** *J Parasitol* 2012, **98:**878–879. doi:10.1645/GE-3098.1. 10.1645/GE-3098.1 22300299

[B13] Lopes AP, Santos H, Neto F, Rodrigues M, Kwok OC, Dubey JP, Cardoso L: **Prevalence of antibodies to*****Toxoplasma gondii*****in dogs from northeastern Portugal.** *J Parasitol* 2011, **97:**418–420. doi:10.1645/GE-2691.1. 10.1645/GE-2691.1 21506866

